# Evaluation of post-traumatic stress disorder (PTSD) and related comorbidities in clinical studies

**DOI:** 10.25122/jml-2022-0120

**Published:** 2022-04

**Authors:** Ioana Anamaria Mureșanu, Diana Alecsandra Grad, Dafin Fior Mureșanu, Stefana-Andrada Dobran, Elian Hapca, Ștefan Strilciuc, Irina Benedek, David Capriș, Bogdan Ovidiu Popescu, Lăcrămioara Perju-Dumbravă, Răzvan Mircea Cherecheș

**Affiliations:** 1. RoNeuro Institute for Neurological Research and Diagnostic, Cluj-Napoca, Romania; 2. Department of Neurosciences, Iuliu Hatieganu University of Medicine and Pharmacy, Cluj-Napoca, Romania; 3. Department of Public Health, Babes-Bolyai University, Cluj-Napoca, Romania; 4. Department of Neuroscience, Carol Davila University of Medicine and Pharmacy, Bucharest, Romania

**Keywords:** post-traumatic stress disorders, TBI, CAPS, post-TBI PTSD, clinical studies, ACT – Acceptance and Commitment Therapy, ASD – Acute Stress Disorder, BDI-II – Beck Depression Inventory 2^nd^ ed, CALM – cognitive applications for life management, CAPS – Clinician-Administered PTSD Scale, CPT – Cognitive Processing Therapy, CPT-C – Cognitive Processing Therapy-Cognitive Only, DASS – Depression Anxiety Stress Scale, D-KEFS – Delis-Kaplan Executive Function System, HADS – Hospital Anxiety and Depression Scale, HIBS – Head Injury Behavior Scale, MBSR – Mindfulness-Based Stress Reduction, MD – Major Depression, NSI – Neurobehavioral Symptom Inventory, OEF – Operation Enduring Freedom, OIF – Operation Iraqi Freedom, OND – Operation New Dawn, PCL-C – Posttraumatic Stress Disorder Checklist Civilian Version, PCL-M – Posttraumatic Stress Disorder Checklist Military Version, PCL-S – Posttraumatic Stress Disorder Checklist Specific Version, PE – prolonged exposure, PTA – post-traumatic amnesia, PTSD – post-traumatic stress disorder, SSRIs – Selective Serotonin re-uptake inhibitors, TAU – trauma-focused group treatment, TBI – Traumatic Brain Injury, THR – Therapeutic horseback riding, WMH – World Mental Health

## Abstract

Patients with traumatic brain injury (TBI) of varying severities are experiencing adverse outcomes during and after rehabilitation. Besides depression and anxiety, post-traumatic stress disorder (PTSD) is highly encountered in civilian and military populations. As more prospective and retrospective studies – focused on evaluating new or old psychological therapies in inpatient, outpatient, or controlled environments, targeting patients with PTSD with or without a history of TBI – are carried out, researchers are employing various scales to measure PTSD as well as other psychiatric diagnoses or cognitive impairments that might appear following TBI. We aimed to explore the literature published between January 2010 and October 2021 by querying three databases. Our preliminary results showed that several scales – such as the Clinician-Administered PTSD Scale (CAPS), the Posttraumatic Stress Disorder Checklist Military Version (PCL-M) as well as Specific Version (PCL-S), and Civilian Version (PCL-C) – have been frequently used for PTSD diagnosis and symptom severity. However, heterogeneity in the scales used when assessing and evaluating additional psychiatric comorbidities and cognitive impairments are due to the study aim and therapeutic approaches. Therefore, conducting an intervention focusing on post-TBI PTSD patients requires increased attention to patients' medical history in capturing multiple cognitive impairments and affected neuropsychological processes when designing the study and including validated instruments for measuring primary and secondary neuropsychological outcomes.

## Introduction

Traumatic brain injury (TBI) is a burdening condition in both acute and chronic phases, with patients exhibiting post-TBI outcomes that affect their overall functioning [1], return to society [2], and inflict a socio-economic burden on their caregivers [3, 4]. It represents a major public health problem that causes over 27 million new cases worldwide within a year, putting tremendous pressure on health systems in low-and middle-income countries (which have the highest-burden regarding the number of cases) and high-income countries alike [5].

Post-TBI, patients (regardless of TBI severity) [6, 7] are at increased risk of developing several psychopathologies, such as anxiety [8], depression [9], and post-traumatic stress disorder (PTSD) [10], among other TBI-related side effects.

In the fifth edition of the Diagnostic and Statistical Manual of Mental Disorders (DSM-V), PTSD is defined by several characteristics grouped under seven major categories, which focus on exposure (to a life-threatening or traumatic event), timing and presence of PTSD symptoms, interaction with PTSD-related triggers, the evolution of adverse effects tied with the traumatic event (on cognition and mood), arousal and reactivity trauma-related alteration, disturbance persistence, as well as disturbance-related consequences on functional impairments (such as occupational and social) and the clear delimitation that the PTSD diagnostic is not caused by medication intake or alcohol consumption [11]. In addition, the prevalence of PTSD cases (reported in community-based studies) diagnosed within the last 6 or 12 months is higher in women than men, and there are more cases diagnosed when the DSM-IV PTSD criteria are used and fewer with the DSM-5 version [12].

In civilian populations, PTSD can be reported following a wide range of traumatic events. WHO World Mental Health (WMH) surveys represent important tools in quantifying mental health impairments in the general population. A 2017 secondary analysis using WHO WMH data reports that trauma was reported by 70.4% of participants (out of the total, 42.7% was due to intimate partner sexual violence, 13.1% was caused by rape, 15.1% by sexual assault, and 9.8% caused by stalking [13]).

The PTSD rates differ in military and civilian populations with a medical history of TBI than those without a TBI. For example, 3.3% more civilians (15.7%) and 26% more military participants (36.8%) were diagnosed with post-TBI PTSD. Post-TBI PTSD was mostly registered among men (61% *vs.* 96%), aged between 35.4 and 31.5 years old, in civilian and military participants [14]. 

Based on the period of the armed conflict, studies show that the lowest percentage of combat diagnosed PTSD is among Gulf War veterans (10%) [15], while the highest among military service personnel who participated in conflicts in Iraq or Afghanistan (13.2%) [16]. 

Recent evidence shows that patients diagnosed only with PTSD or TBI exhibit similar sleep problems (decreased sleep time, continuity, and efficiency – PTSD patients; insomnia, narcolepsy, post-traumatic hypersomnia – TBI patients) [17]. However, TBI severity plays an important factor as higher sleep disturbances are encountered in diagnosed PTSD patients with moderate or severe TBI than in milder TBI cases [18]. Another study on sleep quality comparing cohorts formed of healthy controls, veterans with PTSD with mild TBI, and with PTSD only showed that subjects from the second group reported more often sleepiness during the day (although they were awake less during the night) compared to the other two groups [19].

Cnossen and colleagues conducted a systematic review and meta-analysis on predictors for PTSD and major depression (MD) in patients with TBI history. The authors reported an association between PTSD development and a smaller duration of PTA (post-traumatic amnesia), and remembrance of the event defined as traumatic [20].

A growing body of literature suggests that patients diagnosed with PTSD following a traumatic brain injury can benefit from various pharmacological and psychological therapies. In terms of pharmacological alternatives, selective serotonin re-uptake inhibitors (SSRIs) and tricyclic antidepressants proved to reduce PTSD symptom severity [21], while antipsychotic medicines, such as risperidone and quetiapine, have small positive benefits when included in the treatment regimen [22]. As for psychological interventions, prolonged exposure (PE) and cognitive-processing therapies [23] and meditation-based therapies [24] have positive outcomes.

There are systematic reviews aiming to evaluate, extract and analyze data from articles on the epidemiological type of PTSD [25] on the effect of pharmacological and psychological therapies in patients with PTSD due to TBI [23]. However, other reviews included studies on psychological therapies for PTSD along with other post-TBI pathologies (*i.e.*, anxiety, depression) [21] or have been focusing on a specific therapy [24]. We identified a gap in the literature regarding the presence of a review (literature, narrative, systematic) focusing on scales that measure primary and secondary outcomes as well as those used in PTSD screening for this target group. Therefore, through this brief literature review, we aimed to map instruments used to screen and measure neuropsychological post-TBI status in civilians, veterans, and active service military members who received different therapies.

## Material and methods

We queried three databases for our brief literature review: PubMed, Scopus, and PsycNET. Our search focused on articles published between January 2010 and October 2021. We included quantitative articles published in English that measured PTSD as a primary or secondary study outcome. We excluded articles based on the following criteria: qualitative studies published in another language than English, abstracts, letters to the editor, comments, epidemiological articles, psychological interventions focusing solely on other post-TBI psychological disorders (*i.e.*, anxiety, depression), study protocols, lack of applied scales, lack of abstract, and abstract written in English while the main body in of the article was published in another language. We did not apply a geographical limitation. In order to retrieve articles for our review, we used the following terms: "psychological interventions", "traumatic brain injury", "TBI", "PTSD", "therapy", "psychological treatment", "psychotherapy", "mindfulness", "CBT". For the search strategy conducted on PubMed, we employed MeSH terms. In addition, we used two boolean terms: "AND" was used to narrow the search while "OR" was used to expand the search. The search, abstract, title screening, and data extraction were conducted by the main author (IAM). In the first phase, articles were screened by title and abstract. In the second phase, the full-text screening of the included articles was performed. Data extraction was completed for the remaining articles from the second screening in the third phase.

We extracted data on the listed items: title, author, year, country, university, intervention, sample size, population, inclusion and exclusion criteria, and scales used for screening and measuring neuropsychological outcomes. In addition, several study characteristics (year, country, therapy, and population) were presented descriptively, and other methodological characteristics of study tools usage were briefly summarized.

## Results

Our final selection included seventeen studies, 16 were conducted in the USA and one in the United Kingdom. The sample size ranged from 224 participants for a randomized controlled trial (RCT) involving veterans and their family members to one participant for two case study reports. Our study was primarily based on randomized controlled trials and comparative effectiveness studies. As for the target population, most studies focused on psychological interventions among veterans, and only two included civilians, with one focusing on both veterans and their family members. In addition, studies reported results on several types of therapies: prolonged exposure, Cognitive Processing Therapy (CPT), Cognitive Processing Therapy - Cognitive Only (CPT-C), art therapy, acceptance and commitment therapy, Mindfulness-Based Stress Reduction (MBSR), SMART-CPT, therapeutic horseback riding, and cognitive behavioral therapy (CBT). Table 1 compiles study characteristics. 

**Table 1.** Post-TBI PTSD study characteristics.

**Year**	**Study**	**Country**	**Therapy**	**Study type**	**n studies**	**Population**
**2011**	Chard et al. [26], Alvarez et al. [27]	USA	Cognitive processing therapy – Cognitive only, Cognitive processing therapy (CPT), Present Centered Therapy (PCT)	Comparative effectiveness, efficacy	2	Veterans/active service members; Veterans
**2012**	Wolf et al. [28], Walter, Kiefer & Chard [29]	USA	Prolonged exposure (PE), Cognitive processing therapy Cognitive-Only (CPT-C)	Effectiveness	2	OEF/OIF Veterans; Veterans (OEF/OIF, Persian Gulf War, Vietnam, post-Vietnam)
**2014**	Church&Palmer [30]	USA	Emotional Freedom Techniques	Randomized control trial	1	Veterans
**2015**	Boyd et al. [31], Wolf et al. [32], Cole et al. [33]	USA	Cognitive processing therapy (CPT), Prolonged exposure (PE), Mindfulness-Based Stress Reduction (MBSR)	Case report, Effectiveness study, pilot study	3	Veterans
**2016**	Ragsdale&Horell [34], Strom et al. [35]	USA	CPT, Prolonged exposure (PE)	Comparative effectiveness, case study	2	OEF/OIF/OND veterans; Veterans
**2018**	Ragsdale et al. [36], Johnson et al. [37], Crocker et al. [38], Jak et al. [39],	USA	Exposure therapy (EXP), Prolonged exposure (PE), Therapeutic horseback riding (THR), CPT, SMART-CPT; CPT, SMART-CPT	Comparative effectiveness, randomized wait-list controlled design with repeated measures, clinical trial, randomized clinical trial	4	OIF/OEF/OND veterans; Veterans; OEF/OIF Veterans; OEF/OIF/OND veterans
**2019**	Roche [40], Elbogen et al. [41]	UK, USA	Acceptance and commitment therapy, Art therapy	Case study, randomized clinical trial	2	Civilian hit by a car; Veterans and family members/friends
**2020**	Tanev et al. [42]	USA	Cognitive-behavioral therapy	Naturalistic study	1	OEF/OIF/OND veterans, active-duty service members

The PTSD checklist was used in studies focusing on prolonged exposure (PE) delivery in outpatient and inpatient settings. The first included article on PE had a study sample of ten veterans with a mean age of 33.1 years and medical history of cognitive deficits, PTSD, and TBI. The PCL – military version was used only at two-time points: screening and final session [28]. In 2015, an article with a similar aim evaluated the effect of PE in a sample with the following characteristics: 69 participants (n veterans=51, n active-duty service members=18) with a mean age of 34.01 and having a history of TBI (mild=52, moderate/severe=17). The PTSD checklist PCL was employed at screening and during each session of the intervention [32]. In a different study, the PCL-S version was used before and after the intervention in a sample of 41 veterans (composing two study cohorts: 19 within the PTSD and TBI group, and 22 with PTSD, without a history of TBI), out of which 20 received individual CPT and 21 PE [34]. In each of the abovementioned studies, PE was an effective therapy for veterans with PTSD.

Walter, Kiefer & Chard demonstrated that veterans participating in group and individual CPT-C had decreased PTSD-related symptoms. The sample included 28 veterans with an average age of 36 years and a history of TBI (n mTBI=24, n moderate TBI=4). The PCL – S was used pre-and post-treatment [29].

Cole and colleagues conducted a pilot study based on a pre-post mixed design involving nine veterans (aged 27–58 years) with mild TBI (and an average of 2.1 TBI events per patient), PTSD, and cognitive impairments. Participants were assessed at three-time points: two weeks before and after the start of the intervention as well as three months following completion. Scores for PCL-C highlighted a large effect size both at the immediate post-MBSR (Mindfulness-Based Stress Reduction) and three months post-intervention [33].

Therapeutic horseback riding was used in a randomized wait-list controlled study to assess its effectiveness in reducing PTSD symptoms as well as targeting emotional regulation and loneliness, among others. Assessments were carried out at three-time points (baseline and at weeks 3 and 6). Results showed a statistically significant decline in PCL-M scores at the second and third assessments [37].

A comparison between two groups receiving standard-of-care and emotional freedom techniques (EFT) showed that veterans allocated to the EFT group reported improvements in PTSD symptoms (that were measured at baseline and throughout the study with PCL-M) [30].

Tanev *et al.* investigated if the poor pretreatment cognitive performance and CBT treatment response were associated in a group of 23 veterans and active service members, with a mean age of 32.39, divided into a PTSD-only cohort (n=7) and a PTSD + mild TBI cohort (n=16). They concluded that decreased cognitive ability did not predict poorer CBT results for PTSD symptoms. Participants filled in the checklist before the intervention (baseline), during the intervention (at each CBT session), and post-intervention (1 and 6 months after completing the intervention) [42].

Cognitive processing therapy (CPT) was used in a study by Chard *et al.* involving 47 veterans/active service members. The mean age was 33.93 for participants with mild TBI (n=28) and 38.07 for moderate/severe TBI (n=14). The PCL was administered before and after the CPT intervention [26].

Data on the comparative effectiveness of CPT and trauma-focused group treatment as usual (TAU) analyzed for 207 veterans (n CPT=104, n TAU=93) by Alvarez *et al.* reported that post-discharge veterans from the CPT group exhibit increased symptom improvement compared to TAU. The mean age was 52.23 [27].

Out of 100 veterans included in the study by Jak *et al.*, 51 received SMART-CPT and 49 CPT, with a mean age of 34.39 years, 5.36 years since the last TBI, and on average 2.81 traumatic brain injuries. Both interventions decreased PTSD symptoms, while patients included in the SMART-CPT group exhibited improved cognitive functions involved in learning and attention. The PCL-S instrument was self-administered weekly (12 times) to record the evolution of PTSD symptoms in veterans [39]. Another study using a combination of SMART-CPT and standard CPT employed the PCL-S instrument 12 times during the program and the pre-and post-intervention. The sample consisted of 74 veterans, with a mean age of 34.34 and 2.9 TBI events [38].

Boyd *et al.* used in their case report (focusing on a veteran over 40 years old with mild TBI, cognitive and speech impairments) the PTSD checklist at three-time points (before, during, and after the CPT intervention – which yielded positive results for all three domains of intervention) [31].

CAPS was used in six studies. In two of them, the authors employed this instrument before and after the intervention, while in the rest of the studies, it was used only at one study time point – baseline.

Elbogen *et al.* conducted a randomized control trial on the effect of CALM (cognitive applications for life management) – a cognitive rehabilitation intervention that targeted executive dysfunction and emotional dysregulation, in a sample of 224 participants (out of which 112 suffered a TBI, and the other half were a friend/family member of the patient). The participants' mean age was 36.52, having on average 2.63 TBI events, and 57% suffered a moderate to severe TBI. The trial reported positive results of CALM in decreasing emotional dysregulation [41]. Two case studies by Strom *et al.* followed the evolution of post-TBI PTSD in the context of prolonged exposure (PE) in two war veterans. Their findings are in line with existing literature on the positive effect of PE [41].

The Neurobehavioral Symptom Inventory (NSI) – a tool on neurobehavioral symptoms – was applied in the included studies at the following time points: pre-and post-intervention [29, 31], and at baseline, after the program ended three months following the end of the intervention [38, 39]. Other neurobehavioral tools employed were subscales from the Delis-Kaplan Executive Function System, such as the ones on inhibition/switching and the one on verbal fluency (D-KEFS letter and category fluency), which were employed in by Tanev *et al.* [42] immediately and at six months post-intervention, and by Crocker *et al.* immediately, and three- and six-months post-intervention completion [38].

In the article by Elbogen and colleagues, aside from the D-KEFS Color-Word Inhibition task, the instrument measuring behavioral problems was addressed to caregivers (in contrast with the other two articles). The tool used was the 20-items Head Injury Behavior Scale (HIBS) [41].

Tools employed to quantify anxiety and depression were HADS (Hospital Anxiety and Depression Scale) and the Beck Depression Inventory (2^nd^ ed.; BDI-II).

In a case study by Roche [40], HADS was administered before, after, and at three- and twelve-month follow-up for a cognitive-behavioral approach based on Acceptance and Commitment Therapy (ACT). The patient was a 48-year-old female presenting cognitive difficulties, distress, and trauma symptoms after a car accident.

The Beck Depression Inventory measured depression in seven studies included in this literature review. In five of the studies, the tool was used before and after the intervention [26–28, 31], while in two studies, the scale was used throughout all study sessions [27], with one article using it post-intervention [35].

The BDI-II scale was also used in the retrospective research of Ragsdale and Voss Horell [34] on nonrandomized clinical data to assess the effectiveness of cognitive processing therapy (CPT) or prolonged exposure (PE). The sample consisted of veterans experiencing combat-related PTSD combined with TBI (19 cases) and veterans experiencing only PTSD (22 cases). The sample was mainly composed of males (87.8%), with a mean age of 33 years [34]. In the research of Chard *et al.* [26], BDI-II was used to assess depressive symptoms in 42 veterans completing residential therapy of CPT-C in individual and group settings, while the case study of Boyd *et al.* [31] assessed, among other variables, the variation in depressive symptoms after 12 sessions of Cognitive Processing Therapy–Cognitive Only (CPT-C) on a veteran with PTSD and a history of mild TBI. Furthermore, Strom *et al.* [35] used the BDI-II as well as the Depression Anxiety Stress Scale (DASS) to explore the effects of prolonged exposure (PE), while the research of Crocker [38] used BDI-II, among other scales, to investigate the effects of Cognitive Processing Therapy (CPT) in seventy-four veterans with histories of traumatic brain injuries. Finally, Alvarez *et al.* [27] examined self-reported depression assessed with BDI-II in a quasi-experimental and retrospective cohort study on the effectiveness of group cognitive processing therapy (CPT) compared to trauma-focused group treatment as usual (TAU) in veterans from a residential rehabilitation program.

## Discussion

Our literature review mapped instruments used in 17 studies on post-TBI PTSD, covering several psychological therapeutic approaches such as prolonged exposure, exposure therapy, cognitive processing therapy, mindfulness-based stress reduction, emotional freedom techniques, or acceptance and commitment therapy, among others.

Most studies were conducted in the USA, and veterans mainly formed the analysis cohorts. This can be explained by the high number of American military servicemen and women (in 2018, there were 8.4 million fewer veterans compared to 2,000–26.4 million) who participated in conflicts such as those post-9/11 or the Korean and the Gulf Wars [43]. A 2019 exercise in estimating the absolute number of PTSD and major depression (MD) cases using data from the Uppsala Conflict Database and including countries that experienced war within their geographical borders over twenty-six years (1989–2015) estimated that out of 1.45 billion surviving inhabitants affected by wars, 354 million experienced PTSD (and/or MD), out of which 117 million live with both comorbidities [44]. 

Some of the specific scales employed in civilian and military populations diagnosed post-TBI with PTSD are the Clinician-Administered PTSD Scale (CAPS) and the Posttraumatic Stress Disorder Checklist Military Version (PCL-M) as well as Specific Version (PCL-S), and Civilian Version (PCL-C).

The Clinician-Administered PTSD Scale is a commonly used and validated instrument in establishing a PTSD diagnosis and measuring PTSD-related symptoms in clinical practice and research settings [45]. Our literature review assessed studies that included CAPS versions corresponding to DSM-IV and DSM-V. 

Bryant and colleagues conducted a study enrolling patients from five Australian hospitals, and they compared CAPS DSM-IV (employed at hospital screening and then at assessments at three, twenty-four, and seventy-two months) and CAPS DSM-V (employed only at the final study evaluation – at seventy-two months) for detecting PTSD in patients with acute stress disorder (ASD). Although DSM-5 sensitivity was improved for specific psychiatric pathologies (depression or GAD), the proportions of patients with ASD diagnosed with PTSD were similar when using CAPS DSM-IV or CAPS DSM-V [46].

The latest version reflects the diagnostic criteria listed in DSM-V by adding (three) new symptoms, dropping a criterion, clear delimitation among other group symptoms, and other changes compared to CAPS for DSM-IV [47] and improving screening and scoring processes. The article written by Weathers and colleagues focusing on the psychometric properties of the new edition (which was administered in two cohorts of war veterans) showed that the scale has strong results in terms of interrater and test-retest reliability and internal consistency [45]. 

Another standard measure used for PTSD is the PTSD checklist (PCL) [48], formed of 17 Likert items, with three versions: military (PCL-M), specific trauma (PCL-S), and civilian (PCL-C) [49]. A review focusing on the psychometric properties by Wilkins, Lang & Norman shows that although PCL has internal consistency and strong convergent validity, increased intervals between measure administration can diminish the test-retest parallel. In addition, some of the PCL strengths are related to administration time and difficulty and corresponding measures to DSM criteria. In contrast, PCL limitations are related to reading difficulty, revision and revalidation corresponding to DSM-V, and overestimating the number of PTSD cases correlated with measures for other prevalent psychiatric disorders [49].

Some of the scales included in assessing anxiety and depression are the Hospital Anxiety and Depression Scale (HADS) and The Beck Depression Inventory (2^nd^ ed.; BDI-II).

The Hospital Anxiety and Depression Scale (HADS) [50] represents a self-report measure assessing symptoms of anxiety and depression on a 14-item, four-point scale, with the resulting scores being indicative of the respondents' mood over the past week. It comprises two subscales, one for anxiety and one for depression, scored from 0 to 3, with total scores ranging from 0 to 21 for each scale. When considering the interpretation, higher scores are indicative of more significant distress, and, although there do not seem to be any generally agreed-upon cut off points, the authors suggest considering scores of 8–10 as representative for mild cases, scores of 11–15 as moderate cases and scores of 16 and above as severe ones [50]. HADS is a reliable tool for screening depression and anxiety in TBI patients [51], with sensitivity levels ranging from 62% (depression) to 75% (anxiety), while specificity values were between 69% (anxiety) and 92% (depression) [52].

The Beck Depression Inventory (2^nd^ ed.; BDI-II) was the most used instrument for assessing depression in the context of PTSD and TBI in the studies selected for this literature review.

BDI-II is a self-reporting screening instrument used to assess depressive symptoms within the past two weeks from evaluation. It encompasses 21 statements evaluated on a 4-point scale, with total scores ranging from 0 to 63, and higher scores point to more severe depressive symptoms. The scale presents adequate convergent and discriminant validity, excellent internal consistency, and good test-retest reliability [53, 54].

To measure neurobehavioral symptoms in patients with TBI, several scales are available. One frequently used scale is the Neurobehavioral Symptom Inventory (NSI) which consists of a 22-item self-report questionnaire regarding neurobehavioral symptoms [55]. The first version of the NSI was first published as a symptom checklist in 1995 by Cicerone and Kalmar in the Journal of Head Trauma Rehabilitation. The Department of Veterans Affairs (VA) uses the Neurobehavioral Symptom Inventory (NSI) to measure postconcussive symptoms (present within the last two weeks) in its comprehensive traumatic brain injury (TBI) evaluation; however, the scale domain is not limited to TBI [56]. It is also important to note that most of the research on the NSI was conducted in veteran and military samples with predominantly mild TBI [55]. Nevertheless, studies have demonstrated that this scale is a reliable and valid measure of postconcussive symptoms and helps differentiate veterans with TBI from those without TBI. One downside of this scale is that identifying patients with a history of TBI is strongly influenced by the presence of PTSD, depression, and generalized anxiety, as some analyses on this issue have shown [56, 57].

Another widely used neurobehavioral scale is The Delis-Kaplan Executive Function System (D-KEFS), which consists of a set of standardized tests for comprehensively assessing higher-level cognitive functions (executive functions) in children and adults from the age of 8 to 89. D-KEFS comprises nine tests that measure multiple verbal and non-verbal executive functions. Each test is constructed as a stand-alone instrument and can be administered individually or with other D-KEFS tests based on the specific needs [58]. Heled E. and colleagues demonstrated in their study the superiority of one of the D-KEFS subtests (D-KEFS Sorting Test) in evaluating executive functions of patients that suffered a severe traumatic brain injury and in differentiating TBI patients and healthy controls over two other broadly used tests in evaluating executive functions: The Wisconsin Card Sorting Test and the Trial Making Test. D-KEFS Sorting Test seems to be more sensitive, offers more complexity, and covers more aspects than the previous ones [59]. In a study conducted by Strong *et al.*, another test, the D-KEFS Verbal Fluency subtest, was validated and demonstrated the potential to be clinically helpful in assessing complicated mild-severe TBI [60].

Head Injury Behavior Scale (HIBS) represents another reference scale for evaluating behavioral problems associated with TBI and assessing the level of distress caused by these problems. It consists of two versions, one administered to the patient and another to the caregivers for an optimal evaluation of the burden of TBI-associated behavioral problems. It consists of 20 problems identified from a literature survey on personality changes following TBI. Studies proved that HIBS provides a reliable and valid measure of both caregiver distress and TBI patient distress produced by their behavioral change [61, 62].

Our review shows that, in addition to including tools that diagnose and measure PTSD symptom severity at baseline, it is needed to have multiple usages of these tools across the intervention. Although CAPS or PCL checklists have been employed before and after the intervention (with some authors opting to administer the instruments right after the intervention has ended and some have set different timelines post-intervention), using the tool at each study session or at equally spaced interval delivers a complete symptom evolution which would guide future timing approaches for the patient involved and for patients with similar characteristics.

The limitations of our brief literature review are the following: short period (eleven years), language barriers, the number of queried databases (three), and a limited selection of included therapeutic approaches. The advantages of our brief narrative review are that it mapped, from the included studies, the most used scales from the retrieved studies. However, analyzing single entry tools was difficult due to the increased heterogeneity among included studies.

## Conclusion

Throughout studies targeting psychological interventions that are already being used in clinical practice or evaluating emerging interventions, validated neuropsychological tools for assessing patients are essential in providing a comprehensive clinical psychological picture of patients' evolution. In addition, trialists must pay attention to patients' TBI severity and other psychiatric pathologies (as well as additional comorbidities) when deciding on study design, as the adoption of validated and psychometrically sound instruments is of utter importance.

## Acknowledgments

### Conflict of interest

The authors declare no conflict of interest.

### Authorship

IAM, DAG: conceptualization and methodology. IAM, DAG, SS, SAD, EH, IB, DC, DFM: investigation, data curation, formal analysis. SS, RMC, LPD, BOP, and DFM: supervision and validation. All authors contributed to writing the original draft and reviewing and editing the manuscript.
